# Learning from new colorectal cancers: a qualitative synthesis of significant event reports

**DOI:** 10.3399/BJGPO.2023.0088

**Published:** 2024-04-03

**Authors:** Nicola Cooper-Moss, Achint Bajpai, Neil Smith, Samuel William David Merriel, Umesh Chauhan

**Affiliations:** 1 School of Medicine, Faculty of Health and Biomedical Sciences, University of Central Lancashire, Preston, UK; 2 Centre for Primary Care and Health Services Research, University of Manchester, Manchester, UK; 3 Lancashire and South Cumbria Cancer Alliance, Manchester, UK

**Keywords:** cancer, neoplasms, primary health care, qualitative research

## Abstract

**Background:**

Colorectal cancer is the second leading cause of cancer-related mortality in the UK and a significant contributor to morbidity and mortality worldwide. Early diagnosis provides opportunities for intervention and improved survival. Significant event analysis (SEA) is a well-established quality improvement method for learning from new cancer diagnoses.

**Aim:**

To provide additional insights into diagnostic processes for colorectal cancer and to identify areas for improvement in patient care pathways.

**Design & setting:**

Fifty-three general practices across Pennine Lancashire, England, submitted one or more SEA reports as part of an incentivised scheme.

**Method:**

A standardised data collection form was used to collate learning points and recommendations for improvements. In total, 161 reports were analysed using an inductive framework analysis approach.

**Results:**

There was an overarching theme of building vigilance and collaboration between and within general practices and secondary care. The following four main sub-themes were also identified: education; individualised and flexible care; ownership and continuity; and communication.

**Conclusion:**

These findings provide additional insights into colorectal cancer pathways from a primary care perspective. Practices should be supported in developing protocols for assessment and follow-up of patients with varying presentations. Screening and access to investigations are paramount for improving early diagnosis; however, a flexible diagnostic approach is required according to the individual circumstances of each patient.

## How this fits in

UK survival rates for colorectal cancer remain lower than comparable high-income countries. The incidence of early-onset colorectal cancer is rising, with associated diagnostic delays. This research highlights the complexities of colorectal cancer diagnosis, as patients do not always present with classical symptoms. Findings from multisite SEA should be utilised across primary care networks for peer-to-peer learning and identification of network-wide improvements in colorectal cancer pathways.

## Introduction

Colorectal cancer is a significant contributor to mortality and morbidity among various cancer types. In 2017, colorectal cancer accounted for 11% of new cancer cases, becoming the fourth most common cancer in the UK.^1^ Furthermore, it is the second leading cause of cancer mortality, responsible for 10% of all cancer deaths.^1^ Globally, there were approximately 1.4 million cases of colorectal cancer diagnosed in 2012 and nearly 700 000 deaths; almost two-thirds of which occurred in high-income countries.^2^ From 2010–2014, the 5-year survival rate for colorectal cancer was approximately 60% in the UK.^3^ Survival rates have generally improved across cancer types; however, the UK’s survival rates are considerably lower in comparison with other high-income countries such as Norway, Denmark, and Canada.^3^

Early diagnosis of colorectal cancer is imperative for improving survival. For example, colorectal cancers diagnosed at stage 1 (earliest) have 5-year survival rates over 90%. In contrast, these rates are approximately 10% if diagnosed at stage 4 (latest).^4^ The variation in timing and staging at diagnosis is a recognised factor for the variation in survival rates between countries.^5^ The UK national screening programme for colorectal cancer has been instrumental for improving survival outcomes in asymptomatic patients;^6^ however, primary care continues to hold a central role in the early recognition and referral of patients presenting with a wide range of colorectal cancer symptoms.

Initiatives, such as safety netting and the UK's '2-week wait suspected cancer' referral system, are examples of interventions to improve cancer outcomes. For instance, a cohort study of English general practices reported a lower hazard of death in patients with cancer from practices with high usage of urgent suspect cancer referral pathways.^7^ The 2015–2020 national cancer strategy in England made a specific recommendation that all general practices should undertake significant event analysis (SEA) meetings to learn from new cancer cases, particularly those diagnosed via emergency presentations.^8^

SEA is an approach to learning and quality improvement developed in the mid-1990s in the UK.^9^ Events for analysis could include lapses in safety, administrative incidents, and important clinical events such as a new cancer diagnosis. The analysis involves a team meeting to discuss and critically assess a significant event for learning points and recommendations for improving quality of care. In the UK, SEA is particularly established in primary care, becoming a fundamental element in professional development for GPs and other healthcare professionals.^10^ SEAs in relation to cancer care can examine various components of the care pathway. Areas of strength and weakness can be identified and recommendations at the clinical and organisational levels may help improve quality of care and patient outcomes. The existing literature has primarily focused on learning from SEAs of lung cancer cases^11^ and emergency admissions.^12^ This study aimed to collate colorectal cancer SEAs conducted by multiple practices to highlight the diagnostic pathway for colorectal cancer and identify areas for improvement from a primary care perspective.

## Method

### Setting and practices

General practices across Pennine Lancashire (North West of England) were invited to participate in this study as part of an incentivised local improvement scheme established by the Pennine Lancashire Clinical Commissioning Group (CCG). The area comprised 75 general practices, more than 300 GPs, and included the East Lancashire and Blackburn with Darwen CCGs. The practices cover a patient population of approximately 540 000 and include a range of locations across urban and rural settings. The patient population also varied in socioeconomic and ethnic backgrounds. The region contains pockets of high socioeconomic deprivation and overall colorectal cancer survival is poor compared with national statistics.

### Data collection

Practices were invited to participate by the Pennine Lancashire CCG cancer team. Each practice was asked to search for all new cases of colorectal cancer in the preceding 3 years, between 1 April 2016 and 31 March 2019 using the following electronic Read codes: *B13 Malignant neoplasm of colon* and *B14 Malignant neoplasm of rectum, rectosigmoid junction and anus*. Practices selected a minimum of one colorectal cancer case per 3000 list size to discuss in an in-house SEA meeting. SEAs were completed and submitted electronically to the CCG cancer project manager between 1 April 2019 and 31 March 2020. Any cases of interest were deemed suitable and clinically challenging cases were encouraged to optimise learning.

A standardised electronic SEA template was sent to all practices to document their SEAs. The template was adapted from the Royal College of General Practitioners (RCGP) cancer SEA toolkit^13^ following feedback from local GPs over the previous 3 years. The template comprised of two parts: patient demographics and tables to record discussion outcomes based on learning points; and recommendations for the practice, hospital, and CCG. A sample of the template is available in the supplementary material (appendix). Patient identifiers were not included on the template apart from age and sex, and the local cancer project lead checked their SEA reports to ensure compliance. Practices were pseudo-anonymised by removing any identifying information before analysis. The authors subsequently obtained permission from practices to use any verbatim quotes.

### Data analysis

The data were collated onto an excel spreadsheet. Demographic patient data were summarised with descriptive analysis. NVivo (version 12) software was used for independent thematic analysis by two of the researchers. Different coding frames were used according to the emerging data for each of the recommendation categories: practice, hospital, and CCG. The three coding frames were then amalgamated into common themes. Thematic interpretations were constantly compared with the written quotations and findings were contextually discussed with the project team.

## Results

### Patient demographics and cancer statistics

Fifty-three out of 75 (71%) practices submitted at least one SEA report, resulting in 161 cases included in the analysis (two cases were excluded from the analysis as they involved upper gastrointestinal cancers only). The mean age at diagnosis was 67.7 years (standard deviation [SD] 13.5) and 67% of patients were reported to be alive at the time of the SEA meeting. Table 1 summarises additional descriptive characteristics.

**Table 1.** Summary of patient demographics and cancer statistics (*n* = 161)

Characteristic	*n* (%)
**Sex**	
Male	86 (53.4)
**Screening status**	
Positive	14 (8.7)
Negative	25 (15.5)
Unknown	33 (20.5)
Ineligible for routine screening	89 (55.3)
**Type of tumour**	
Colon	107 (66.4)
Anorectal only	51 (31.7)
Not reported	3 (1.9)
**Node involvement at diagnosis**	
Present	72 (44.7)
**Metastasis at diagnosis**	
Present	27 (16.8)
**Diagnosis and survival statistics – mean (SD)**	
Mean age (SD) at time of diagnosis	67.7 (13.5)
Days from last bowel screening to diagnosis	544.5 (632.5)
Days from diagnosis to death	327.1 (343.5)

SD = standard deviation.

### Thematic analysis

#### Overview of main themes

**Figure 1.**
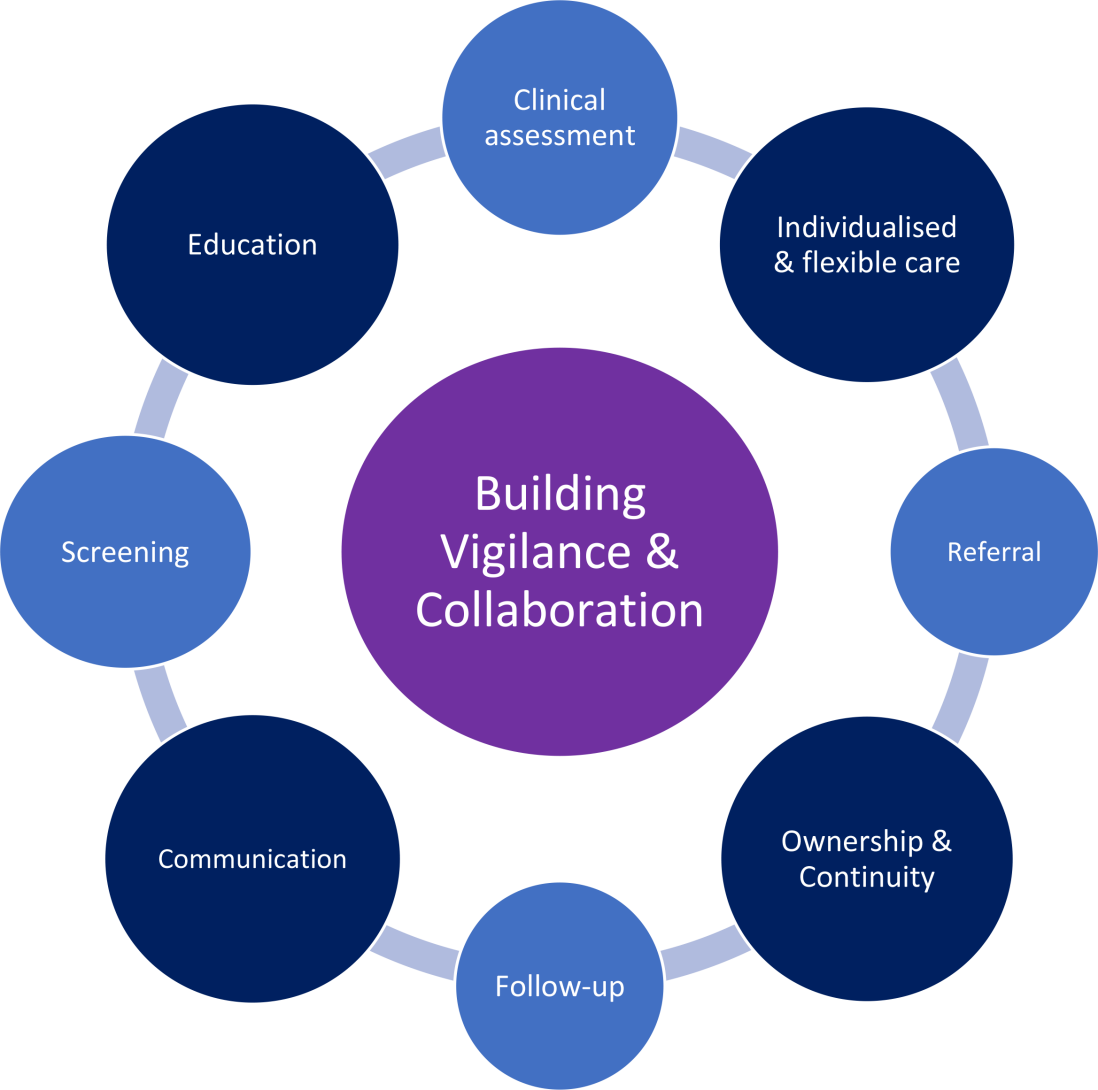
Summary of main themes and the patient care pathway

The primary theme connecting the SEAs was building vigilance and collaboration between and within general practices, secondary care, and CCGs. Building a vigilant and collaborative approach was highlighted throughout all aspects of the patient care pathway including screening, clinical assessment, referral, and follow-up. Four main sub-themes were also derived from the data. These were as follows: education; individualised and flexible care; ownership and continuity; and communication. An overview of the themes is depicted in Figure 1 and each theme is described further below.

#### Building vigilance and collaboration

**Figure 2.**
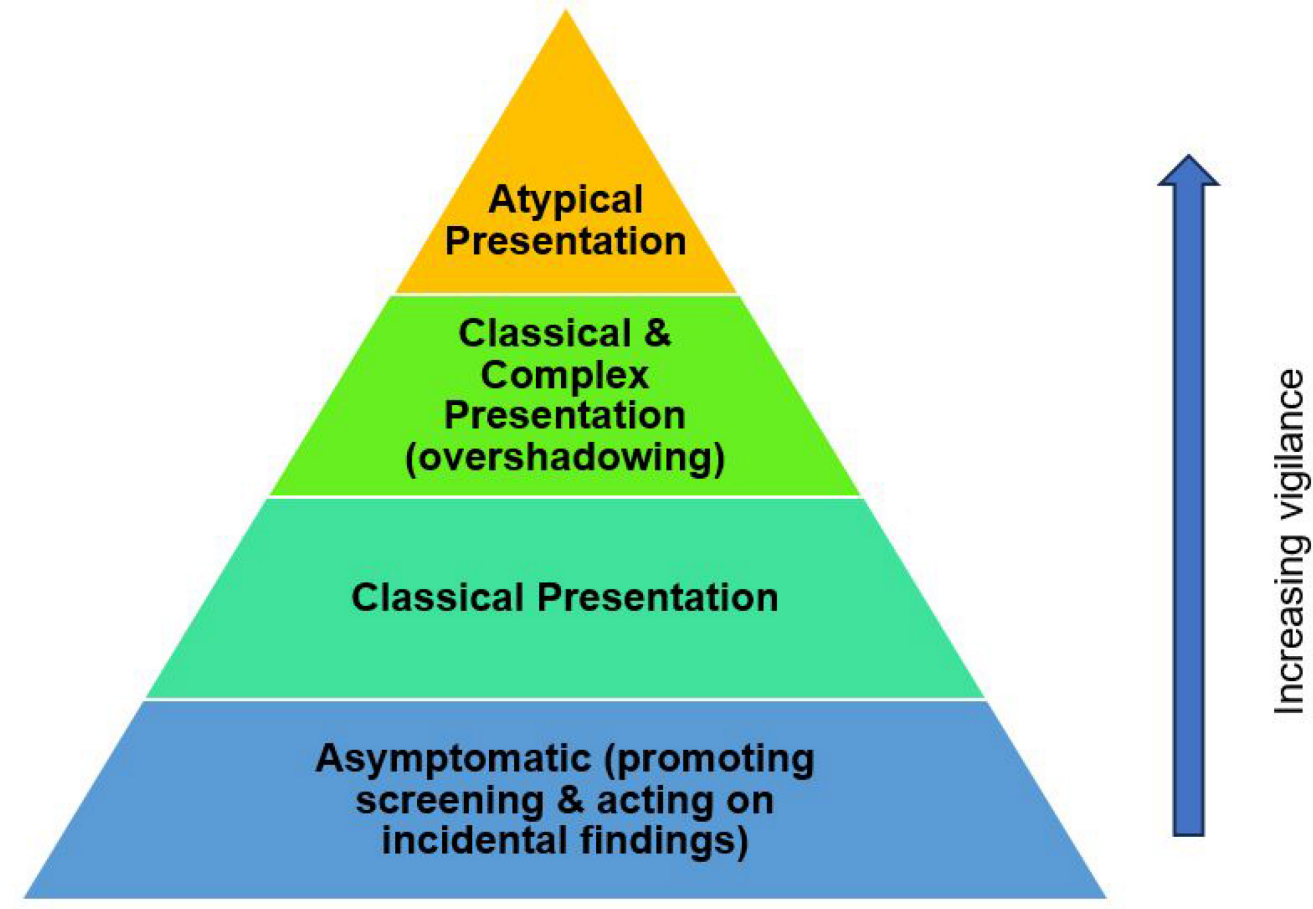
Vigilance pyramid for presentations of bowel cancer

GPs frequently recognised classical symptom patterns associated with colorectal cancer; however, the concept of building vigilance combines the necessity of awareness and practising caution with the wider range of complex and atypical presentations. Various levels of vigilance are required according to the type of clinical presentation, which can be grouped into four categories: (1) asymptomatic (incidental or screening detected); (2) classical presentations; (3) classical and complex presentations; and (4) atypical presentations. It is considered that each group of presentations requires an increased level of vigilance to broaden the diagnostic net for colorectal cancer, illustrated by the ‘vigilance pyramid’ in Figure 2. Classical presentations relate to the typical red flag symptoms of colorectal cancer, such as unexplained rectal bleeding or persistent changes in bowel habit. However, patients with classical and complex presentations may have obvious symptoms, yet diagnosis is complicated by multimorbidity or diagnostic overshadowing from other conditions such as haemorrhoids or presumed intentional weight loss. Atypical presentations were reported in younger patients, and those presenting with non-specific symptoms and/or negative screening results:

*'Continue to be vigilant with changes in bowel habit … don’t be reassured by piles.'* (Practice 34)

*'Bowel cancer affects younger patients as well. Patients can feel fairly well/work even with metastatic life-threatening cancer.'* (Practice 2)

*'We correctly identified an iron deficiency picture on a blood test taken for routine chronic disease monitoring. This led to a rapid assessment and referral followed by surgery with a good chance of cure.'* (Practice 44)

'*Remember to do the FIT* [faecal immunochemical test] *in patients with vague abdominal symptoms*.' (Practice 8)

*'Our patients were all younger than 60 years of age, the youngest being 38. All were women. CA125 can be positive in bowel cancer. Continue to have regular team meetings to share learning.'* (Practice 30)

Building collaboration also functions similarly by acknowledging the need to improve existing links between and within primary and secondary care. GPs and colleagues outside of primary care already work together in the diagnostic pathway for bowel cancer; however, SEA discussions suggested areas for improvement and therefore there is a need to build on the current level of collaboration:

'*Recommend that all cancer champions* [in primary care] *are happy to be contacted by secondary care.*' (Practice 25)

'*Secondary care to provide quick answers/information when advice requested*.' (Practice 60)

'*Ensure all correspondence is relayed to GPs to allow the GP to have a better picture of the patient journey.'* (Practice 7)

'*Complete primary care investigations before or at time of referrals to speed things up.*' (Practice 73)

*'Continue to provide "hot clinic" access for patients with concerning symptoms.'* (Practice 89)

#### Education

Education involving both patients and clinicians frequently featured throughout the SEA reports. Education for patients was considered in the form of health promotion on the importance of bowel cancer screening participation and early presentation with red flag symptoms. The educational value of learning from new cases in the form of practice SEA meetings was also widely recognised:

*'Funding support to improve patient education/awareness of cancer symptoms* [CCG recommendation]*.'* (Practice 2)

*'Advertise and talk about the importance of bowel screening.'* (Practice 13)

*'Ensure that the learning from the SEA is shared with the wider team including locum doctors.'* (Practice 30)

'*Run educational events on early cancer diagnosis* [CCG recommendation]*.'* (Practice 44)

*'Continue to audit cancer diagnoses (referral route, stage at diagnosis, pathway, referral, outcomes).'* (Practice 60)

#### Individualised and flexible care

Individualised and flexible care approaches were widely appreciated to optimise opportunities for early diagnosis and quality of care for patients with bowel cancer. This included remaining vigilant to recognise complex and atypical presentations, while adopting flexible shared decision-making approaches:

*'Using shared decision making to help the family and patient reach a decision based on the best balance of risk/benefits.'* (Practice 11)

*'Allow 2-week referrals even if the patient is already under another oncologist.'* (Practice 65)

*'Bowel cancer can present vaguely, have some flexibility on referral criteria.'* (Practice 31)

#### Ownership and continuity

Ownership and continuity were considered imperative for minimising missed opportunities for early diagnosis. This largely related to the main theme of building vigilance by ensuring re-assessment of patients with unexplained persistent symptoms, and prompt review of investigation results. This theme was also prominent in the recommendations made for improving transitional care between primary and secondary care, and follow-up processes within secondary care. These recommendations included confirming urgent suspected cancer referrals, follow-up of non-attenders, and overseeing ongoing care following diagnosis:

*'Ensure clinicians follow the patient through at least the initial diagnosis journey.'* (Practice 35)

*'Re-assess and re-examine patients presenting again irrespective of previous consultations.'* (Practice 20)

*'When symptoms persist consider repeating the bloods. In this patient though the bloods were initially normal on repeating the bloods they showed iron deficiency anaemia with thrombocytosis.'* (Practice 6)

*'Review patients again if symptoms fail to improve.'* (Practice 54)

*'Ensure results are communicated to patients properly by clinicians especially in a time when there is increased turnover of clinicians, so sometimes there may be gaps in the continuity of care in GP and trust.'* (Practice 12)

*'GP to be more explicit as to the possible diagnosis and reiterate the importance of attending hospital appointments.*' (Practice 25)

*'No follow-up from hospital should be chased as patient may have been lost in system, just do not think its due to winter pressures or holidays.'* (Practice 52)

#### Communication

Communication was repeatedly mentioned in the contexts of both interactions with patients and interdisciplinary working, thus relating to the main theme of building collaboration. Secondary care was considered to have an important role in ensuring that diagnoses and management plans are explained clearly to patients and GPs. In addition, discharge letters and investigation results need to be sent and reviewed in a timely fashion:

*'Promote discussion and reflection between primary and secondary care … good communication between patients and clinicians helps to provide a more positive experience* [for patients].' (Practice 26)

*'Provide a clear summary of new cancer diagnosis with important details that a GP might need.*' (Practice 89)

*'Communicate in a timely manner — these patients are often having multiple tests/frequent reviews but in some instances, it can take weeks for letters to arrive.'* (Practice 2)

*'Ensure results are communicated to patients properly by clinicians especially in a time when increased turnover of clinicians.'* (Practice 12)

*'Communication from the OOHs* [out of hours] *service to the GP needs to include the urgency of any follow-up.'* (Practice 27)

### Learning and recommendations for practices, hospitals, and commissioners

Factors related to early diagnosis in primary care were frequently discussed, specifically concerning presentation, clinical assessment, and diagnostic overshadowing. Practices reported an increased awareness of 'red flag' symptoms, such as rectal bleeding and iron deficiency anaemia, following the SEA discussions. Atypical presentations were also mentioned, with caution for patients who may present with vague or few red flag symptoms. In addition, recommendations for observing younger patients were noted. Clinical assessment focused on the importance of rectal examination, family history, and a review of relevant investigations. Diagnostic overshadowing was a concern shown through discussion of haemorrhoids and presumed intentional weight loss.

The risk of false assurance through negative investigation and screening results was evident in the SEA discussions. A lower threshold for the use of the faecal immunochemical test (FIT) was recommended, particularly for patients with atypical symptoms. The diagnostic limitations of computed tomography (CT) scans were recognised; however, direct access for GPs was advocated in the context of diagnostic uncertainty or for patients declining referral for colonoscopy. Table 2 summarises the diagnostic factors for colorectal cancer that GPs should consider. Verbatim quotes are included to provide further context.

**Table 2.** Diagnostic factors and relevant verbatim quotes for bowel cancer in primary care

Red flags		Atypical presentations	
Change in bowel habit Rectal bleeding Iron deficiency anaemia Thrombocytosis Decreased appetite or weight	*'Rectal bleeding needs to be taken seriously and investigated quickly, especially in someone aged 50+*.' (Practice 41) *'Always investigate unexplained anaemia.'*(Practice 14)	Negative screening results Lack of 'classic' symptoms Unchanged bowel habit Younger patients	*'Patient could have underlying significant illness even though they present asymptomatic*.'(Practice 50) *'We are seeing bowel cancer in young age and even though they don’t present with classical symptoms we should keep sinister pathology in mind*.' (Practice 52)
**Clinical assessment**		**Diagnostic overshadowing**	
History of symptoms Family history Abdominal and rectal exam Screening status	*'Always ask about family history.'* (Practice 28)‘ *The importance of a PR examination in all patients with PR symptoms.'* (Practice 57)	Haemorrhoids Intentional weight loss Multimorbidity Repeat presentations	*'Consider possibility of anal cancer when examining possible haemorrhoids*.' (Practice 75)*'Take repeated presentations seriously*.' (Practice 28)

PR = per rectal.

Recommendations for hospitals focused on reduced radiology reporting times, flexibility with suspected cancer referrals, ownership of patient follow-up, and improved communication with patients and primary care, as previously described. Concerning referrals, areas of improvement applied to increasing the flexibility for processing 2-week suspected cancer referrals from primary care and referrals that involve multiple hospital departments:

'*Show understanding of the diagnostic uncertainty in primary care, particularly where a 2-week wait referral does not strictly meeting guidelines … allow more advice calls or letters which might prevent a referral where there is great uncertainty*.' (Practice 40)

*'Ensure clinicians take ownership of test results. These should be actioned by the clinician/department requesting the test.*' (Practice 35)

Comissioners were recommended to support the promotion of colorectal cancer screening and implementation of standardised systems for follow-up of non-responders. Commissioners were also urged to continue support of multisite SEA and facilitate shared learning across practices. Another area for education included multidisciplinary cancer education events to highlight best practices (previously highlighted by the education theme). Commissioners were also asked to oversee effective communication between primary and secondary care and improve flexibility with suspected cancer referrals by updating referral protocols. Maintaining primary care access to hospital radiological investigations such as CT scans was an additional point of discussion:

'*Practices should be supported to chase up patients who don’t attend for bowel screening and patients who present with cancer symptoms below the age for screening.*' (Practice 5)

*'Ensure practices are following best practice to ensure that all 2-week wait referrals are coded and followed up.'* (Practice 40)

'*Continue providing access to CT scans for GPs as this is likely to save lives.*' (Practice 6)

## Discussion

### Summary

The findings of this study confirm and provide additional insights into colorectal cancer pathways from the perspectives of primary care professionals. This thematic analysis has elicited clear learning points and recommendations at different levels of primary and secondary care for the colorectal cancer diagnostic pathway. Building vigilance and collaboration throughout the patient care pathway is essential for reducing missed opportunities in colorectal cancer diagnosis and improving care quality.

### Strengths and limitations

The use of multisite cancer-specific SEA is well established in Pennine Lancashire, accounting for the excellent response rate. While it is plausable that non-participating practices could be systematically different from those that submitted SEA reports, there were a high number of practices participating (71%) and large number of SEAs reviewed. Anonymisation of practice identifying information and independent thematic analysis between two authors minimised potential confirmation bias. The reflective nature of SEA produced data providing a holistic picture of cases including relevant pre-diagnosis factors such as comorbidities or the influence of prior bowel screening outcomes. Member checking for verbatim quotes has further served to validate the analysis findings.

A baseline level of engagement was presumed for all practices that submitted cases and included details of their SEA discussions; however, it is uncertain whether the submissions were truly representative of what was discussed in the SEA meetings. Conversely, SEA reports provide a practical means for collecting specific insights into real-life team discussions and multisite recommendations. The patient demographic data were incomplete for some submitted cases. However, such data were not intended to influence the thematic analysis, rather to provide a crude overview of the types of cases selected for SEA.

Clinicians may have chosen certain memorable cases owing to factors such as their atypical presentation or emotional impact. The study team considered an alternative standardised approach to case selection; however, this approach was considered fitting to the flexible and educational nature of the SEA process. Generalisability of results is also cautioned owing to the demographics of the patient sample. The mean age at diagnosis for patients in this study was 67.7 years (SD 13.5), which is slightly lower than the mean age of 69.4 years (SD 11.2) reported in the UK for 2014.^14^ Furthermore, the areas covered by participating practices include those with high levels of deprivation.^15^ The authors consider this to be a strength of the study, as these populations are often underserved in research.

### Comparison with existing literature

SEA was traditionally applied in healthcare settings to learn from adverse incidents and near misses. However, recent years have shown a greater focus on the use of SEA as an educational quality improvement activity, focusing on aspects of patient care that are considered significant for patients, such as a new cancer diagnosis.^16^ It is conceivable that the dynamics of the SEA discussions could vary according to how SEA is subjectively viewed and applied by individual clinicians; however, shared learning across practices encourages a more objective assessment of circumstances. This study is the largest to our knowledge to assess learning from SEAs performed in the context of colorectal cancer. A previous analysis of SEAs in cancers diagnosed at emergency presentation also used thematic analysis to determine learning points and recommendations, however, only 21 out of 222 (9.4%) cases involved colorectal cancer, and the analysis was not specific to colorectal cancer.^12^

A cohort study assessing healthcare use leading up to a colorectal cancer diagnosis (*n* = 943) found that a majority (84%) of emergency-presentation patients went to their GP within 6 months of diagnosis.^17^ These patients presented with fewer symptoms that would have met the urgent referral criteria in comparison with patients who were diagnosed after referral by a GP.^17^ Another prospective study of 1606 patients also assessed differences in emergency and non-emergency presentations. Both groups had similar patterns in primary care consultations at a background level (up to 5 years before diagnosis) and closer to diagnosis (within 1 year).^18^ However, emergency-presenting patients demonstrated fewer relevant symptoms of colorectal cancer compared with non-emergency patients. Furthermore, any red flag symptoms, such as rectal bleeding, were recorded less frequently; however, around 20% of emergency presenters did have such symptoms in the year leading up to diagnosis.^18^ These studies indicate clear differences in presentation between emergency and GP-referred diagnoses, which are consistent with the major theme of building vigilance and appreciating the impact of atypical presentation. A study assessing mortality between emergency and GP-referred cancer diagnoses found greater short-term mortality in the former, with an excess mortality ratio of 5.9 for colon cancer 1 month after diagnosis.^19^ Therefore, it is paramount to recognise opportunities in primary care to improve early diagnosis.

The SEA reports frequently highlighted the importance of re-assessment in patients with persistent or evolving symptoms. Some of the SEAs specified the importance of continuity of care with the same clinician, whereas others made more generic statements that related to vigilant diagnostic approaches adopted by any member of the clinical team. The role of continuity is similarly mixed within the existing literature, suggesting continuity of care may be associated with different times to diagnosis depending on its relation to when the initial symptoms are recorded, that is, the index consultation. For example, a retrospective analysis of consultations up to 24 months before diagnosis found that continuity of doctor before the index consultation was associated with a later diagnosis of colorectal cancer, whereas continuity after the index consultation was associated with an earlier diagnosis.^20^ A study linking data from the GP experience survey also suggests that confidence and trust in the doctor may be more important for early cancer diagnosis than ease of access or choice of doctor.^21^ This therefore highlights the importance of considering continuity in the context of wider factors influencing the doctor–patient relationship and clinical decision making.

### Implications for practice and research

A diagnostic approach grounded on building vigilance and collaboration is essential to account for diagnostic factors that may delay a colorectal cancer diagnosis. This approach must account for individualised patient-specific factors, as classical presentations of colorectal cancer may not manifest in all patients. Utilisation of screening and appropriate investigations is another important aspect of diagnosis. For example, the frequent recommendations for using the FIT test are consistent with the DG30 guideline from the National Institute for Health and Care Excellence (NICE), which recommends requesting FIT for patients with symptoms that are not necessarily red flag in nature.^22^ A recent NICE guidance update draft also encourages the use of FIT to inform referral decisions, emphasising the importance of adequate safety netting and follow-up, in keeping with the themes of communication and ownership and continuity found in this study.^23^

Flexibility with 2-week cancer referrals may allow for greater support for circumstances where the referral criteria do not fully satisfy the referral guidelines. A review of cancer diagnoses in primary care highlights the complexity of the referral decision faced by GPs when seeing patients with ambiguous presentations that do not meet referral criteria but warrant a referral nonetheless.^24^ For instance, patients with milder forms of anaemia do not necessarily have a negligible risk of colorectal cancer, especially if they present with ambiguous or atypical symptoms. The need for a flexible diagnostic approach to non-specific cancer symptoms also fits with previous studies that highlight the importance of acting on clinical suspicions or 'gut feelings'.^25,26^ This also fits with the current drive for primary care networks (PCNs) to improve early cancer diagnosis through development of non-specific symptom pathways.^27^

The broad range of cases from multisite SEA should be utilised across PCNs for peer-to-peer learning and identification of network-wide improvements in cancer pathways. SEAs for other cancer types may allow for more holistic improvements in cancer pathways where appropriate. Further research is required regarding the use of SEA for improving and sustaining cancer outcomes, and the factors influencing system-level change.
